# Nitrosopersulfide (SSNO^−^) accounts for sustained NO bioactivity of S-nitrosothiols following reaction with sulfide^☆^

**DOI:** 10.1016/j.redox.2013.12.031

**Published:** 2014-01-11

**Authors:** Miriam M. Cortese-Krott, Bernadette O. Fernandez, José L.T. Santos, Evanthia Mergia, Marian Grman, Péter Nagy, Malte Kelm, Anthony Butler, Martin Feelisch

**Affiliations:** a Cardiovascular Research Laboratory, Department of Cardiology, Pneumology and Angiology, Medical Faculty, Heinrich Heine University of Düsseldorf, Düsseldorf, Germany; b Clinical and Experimental Sciences, Faculty of Medicine, University of Southampton, Southampton General Hospital, Tremona Road, Southampton, UK; c Institute for Pharmacology and Toxicology, Ruhr-University Bochum, Bochum, Germany; d Institute of Molecular Physiology and Genetics, Slovak Academy of Sciences, Bratislava, Slovak Republic; e Department of Molecular Immunology and Toxicology, National Institute of Oncology, Ráth György utca 7-9, Budapest, Hungary; f Medical School, University of St-Andrews, St-Andrews, Fife, Scotland

**Keywords:** DMF, dimetylformamide, DMSO, dimethylsulfoxide, CysNO, S-nitrosocysteine, GSNO, S-nitrosoglutathione, IPN, isopentyl nitrite, NO, nitric oxide, NO^+^, nitrosonium, SSNO^−^, nitrosopersulfide, perthionitrite, PDE, phopsphodiesterase, RFL-6, rat fibroblastoid-like cell line, sGC, soluble guanylyl cyclase, SNO^−^, thionitrite, SNAP, S-nitrosopenicillamine, Hydrogen sulfide, Nitric oxide, Polysulfides, cGMP, HSNO, Nitroxyl

## Abstract

Sulfide salts are known to promote the release of nitric oxide (NO) from S-nitrosothiols and potentiate their vasorelaxant activity, but much of the cross-talk between hydrogen sulfide and NO is believed to occur via functional interactions of cell regulatory elements such as phosphodiesterases. Using RFL-6 cells as an NO reporter system we sought to investigate whether sulfide can also modulate nitrosothiol-mediated soluble guanylyl cyclase (sGC) activation following direct chemical interaction. We find a U-shaped dose response relationship where low sulfide concentrations attenuate sGC stimulation by S-nitrosopenicillamine (SNAP) and cyclic GMP levels are restored at equimolar ratios. Similar results are observed when intracellular sulfide levels are raised by pre-incubation with the sulfide donor, GYY4137. The outcome of direct sulfide/nitrosothiol interactions also critically depends on molar reactant ratios and is accompanied by oxygen consumption. With sulfide in excess, a ‘yellow compound’ accumulates that is indistinguishable from the product of solid-phase transnitrosation of either hydrosulfide or hydrodisulfide and assigned to be nitrosopersulfide (perthionitrite, SSNO^−^; *λ*_max_ 412 nm in aqueous buffers, pH 7.4; 448 nm in DMF). Time-resolved chemiluminescence and UV–visible spectroscopy analyses suggest that its generation is preceded by formation of the short-lived NO-donor, thionitrite (SNO^−^). In contrast to the latter, SSNO^−^ is rather stable at physiological pH and generates both NO and polysulfides on decomposition, resulting in sustained potentiation of SNAP-induced sGC stimulation. Thus, sulfide reacts with nitrosothiols to form multiple bioactive products; SSNO^−^ rather than SNO^−^ may account for some of the longer-lived effects of nitrosothiols and contribute to sulfide and NO signaling.

## Highlights

• Sulfide modulates the bioactivity of nitrosothiols in a concentration-dependent manner.
• Nitrosopersulfide anions (SSNO^−^) accumulate at high sulfide/RSNO ratios.
• SSNO^−^ releases NO and is surprisingly stable in the presence of reduced thiols.
• SSNO^−^ is a potent activator of soluble guanylyl cyclase.
• SSNO^−^ is likely to contribute to NO and hydrogen sulfide/polysulfide signaling.

## Graphical abstract

**Figure:**
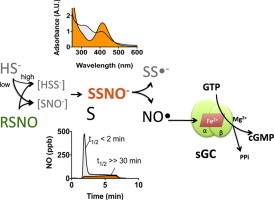

## Introduction

Hydrogen sulfide (H_2_S), known as a noxious malodorous gas of volcanic and biogenic origin for centuries, has recently been shown to exert a multitude of beneficial biological effects, some of which have therapeutic potential [1], [2]. Pharmacological doses of simple sulfide salts have been demonstrated to protect tissues against ischemia-reperfusion injury [3], [4]. Endogenous sulfide production appears to be involved in the physiological modulation of numerous cellular processes in almost every organ system, including control of vascular tone and blood pressure regulation [4]. It has been suggested that H_2_S should be recognized as a third ‘gasotransmitter’, alongside nitric oxide (NO) and carbon monoxide [5], [6], although this is not unanimously accepted [7].

In physiological systems, only a small part of H_2_S actually exists in the form of dissolved gas. As a diprotic weak acid (mean p*Ka*_1_=7.0 and p*Ka*_2_>12 at 25 °C [8]), H_2_S rapidly deprotonates to form hydrosulfide anions (HS^−^) with negligible amounts of S^2−^ existing at physiological pH [8]. The relative amounts of the three species at equilibrium depend on temperature, pH, ionic strength, amount of H_2_S gas leaving the solution, as well as “side” reactions including sulfide oxidation to form sulfite, sulfate, and thiosulfate, and polymerization reactions generating polysulfides and polythionates. These products themselves are characterized by complex ionization equilibria which may affect solution pH and thus concentrations of HS^−^ and dissolved H_2_S [8], [9]. For simplicity, all three forms co-existing in equilibrium (H_2_S, HS^−^, and S^2−^) will hereinafter be referred to as ‘sulfide’.

Striking similarities between some of the effects of NO and sulfide, in particular with regard to their role in control of vascular tone and blood pressure regulation, together with interesting mutual regulatory effects, raised the possibility of a cross-talk between these species [10], [11], [12], [13], [14], [15], [16], [17], [18]. Peculiarly, pharmacological application of sulfide was shown to induce both vasodilation [19] and vasoconstriction [10] *ex vivo*, and both *hypo*tensive and *hyper*tensive effects have been described in vivo [10], [19]. Wang surmised that the effects of sulfide may depend on the specific vascular bed (conductance vs. resistance vessels), cell type (endothelial vs. smooth muscle cells), concentration of sulfide and presence or absence of NO/nitric oxide synthase in a particular experimental setting [20]. Moore et al. [10], [21], [22] proposed that vasoconstriction and hypertensive effects of lower sulfide doses were due to a direct chemical interaction between vascular NO and sulfide leading the formation of an intermediate, which was susceptible to destruction by addition of transition metals. These authors hypothesized that this intermediate might be a “nitrosothiol”, probably thionitrous acid (HSNO) [21].

HSNO was described by Williams as the obvious product of sulfide nitrosation [23]; likewise, HSNO is the obvious product of the reaction between HS^−^ and nitrosothiols. Indeed, a recent report suggests that HSNO is formed by direct reaction of S-nitrosoglutathione (GSNO) with sulfide in phosphate buffer at pH 7, and is stable enough to transport NO intermediates across cell membranes [24]. In contrast, earlier reports found this compound to be unstable and reactive, necessitating characterization of its isomerization properties to be carried out in a low temperature argon matrix [25].

The reaction between sulfide and S-nitrosothiols has been shown to promote NO release, potentiating their vasorelaxant activity in aortic rings [26] and allowing for nitrosothiol quantification by gas phase chemiluminescence [27]. It is not clear at present whether these effects of sulfide are due to post-translational modification of vascular proteins, accelerated nitrosothiol decomposition, formation of NO-generating intermediates, or a combination of these putative mechanisms [17], [18]. Independent of the above chemical studies, a recent report suggests that much of the H_2_S/NO cross-talk might occur by modulation of cyclic nucleotide breakdown following inhibition of phosphodiesterase (PDE) activity [28].

The aim of the present study was to investigate whether sulfide can modulate S-nitrosothiol-mediated activation of the NO receptor soluble guanylyl cyclase (sGC) in a simple cellular system that lacks an endogenous NO production machinery and expresses low levels of PDE5 [29]. We found that sulfide modulates nitrosothiol bioactivity in a concentration-dependent manner. Under conditions of excess sulfide, a ‘yellow compound’ accumulates that has the potential to spontaneously release NO and activate sGC. We assign this species to be nitrosopersulfide (SSNO^−^) and propose that this reaction product, rather than HSNO, is the main carrier of sustained NO bioactivity following interaction of sulfide with nitrosothiols.

## Materials and methods

### Materials

Ultrapure water (Milli-Q, Millipore), S-nitroso-N-acetyl-DL-penicillamine (SNAP), sodium persulfide (Na_2_S_2_, sodium disulfide; Sage Chemical Co., Ltd, Hangzhou, China), p-methoxyphenyl-morpholino-phosphinodithioic acid (GYY4137), 3-isobutyl-1-methylxanthine (IBMX) and 3'-methoxy-3-oxo-3H-spiro[isobenzofuran-1,9'-xanthen]-6'-yl 2-(pyridin-2-yldisulfanyl)benzoate (Washington State Probe-1, WSP-1) from Cayman Chemicals (Biomol, Hamburg, Germany) was used. Unless otherwise specified, all other chemicals were of the highest purity available and purchased from Sigma-Aldrich (Schnelldorf, Germany or Gillingham, Dorset, UK), cell culture plastics from Greiner (Frickenhausen, Germany), and other cell culture material from PAA (Pashing, Austria). Fetal bovine serum (FBS) was from Cambrex (Lonza, Cologne, Germany).

### Cell culture

Rat fibroblastoid-like (RFL-6, ATCC CCL192™) cells were purchased from LGC Standards GmbH (Wesel, Germany) and cultured from passage 8–18 in T9 flasks using RPMI 1640, supplemented with 20% fetal bovine serum and antibiotics in a CO_2_ incubator under standard cell culture conditions.

### Preparation of stock solutions and RSNO/sulfide mixtures

Stock solutions of SNAP (100 mM, DMSO or DMF), IBMX (50 mM, DMSO), GYY4137 stocks (40 mM, DMSO), and WSP-1 (5 mM, DMSO) were kept aliquoted at −20 °C until use. Stock solutions of S-nitrosocysteine (CysNO) and S-nitrosoglutathione (GSNO) were freshly prepared via reaction of the reduced thiols with acidified nitrite [30] and used immediately. Na_2_S stock solutions (200 mM) were prepared fresh before each experiment by dissolving anhydrous Na_2_S in a strong buffer (TRIS or phosphate buffer 1 M at pH 7.4) and diluted further in 100 mM TRIS or phosphate buffer pH 7.4 immediately before use. Incubation mixtures of nitrosothiols with Na_2_S were obtained by adding appropriate volumes of the stock solutions directly to the incubation buffer to achieve final concentrations of 1 mM for the nitrosothiol and 0.2–10 mM for sulfide, as indicated in text and figures.

### Measurements of sGC activation by sulfide, SNAP, and its mixtures

**Fig. 1:**
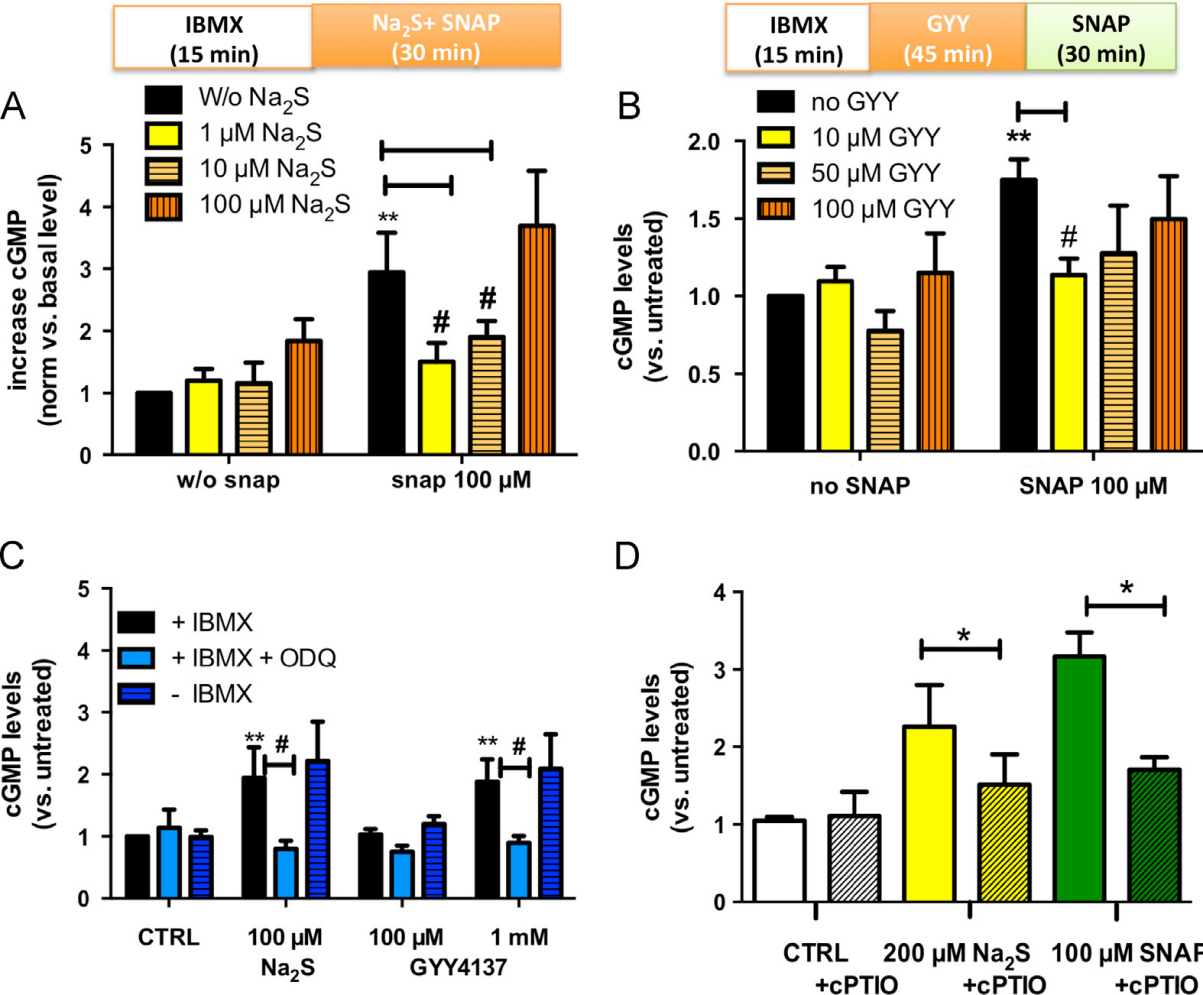
Sulfide modulates s-nitrosothiol bioactivity in a concentration-dependent manner. (A) Changes in cGMP concentration after treatment of RFL-6 cells with Na_2_S, SNAP, or their combination (*n*=5). (B) Effects of SNAP on cells pre-incubated with the sulfide donor GYY4137 (*n*=4). (C) Effects of sGC and PDE inhibition on Na_2_S and GYY4137 induced activation of sGC (*n*=4). (D) Effects of cPTIO on sulfide and SNAP induced sGC activation (*n*=3) ^⁎^*p*<0.05; ^⁎⁎^*p*<0.01 vs. CTRL, # *p*<0.05, *T*-test.

sGC activity was estimated by measurement of intracellular cGMP levels of RFL-6 reporter cells as described [31], with minor modifications. Briefly, 1.5×10^5^ cells/well were seeded into 6-well plates, grown for 48 h until confluence, washed twice with 1 ml phosphate buffered saline (PBS; pre-warmed to 37 °C), and pre-treated for 15 min with the PDE inhibitor IBMX (500 µM) in culture medium without serum (treatment medium). After washing with PBS, 1 ml fresh treatment medium was added and cells were then treated as indicated. In this system, SNAP and other NO-donors cause concentration-dependent increases in intracellular cGMP, which are fully inhibited by NO-scavengers (Fig. 1 and Fig. S1). To capture a wide-enough time window for NO bioactivity measurements, all SNAP/sulfide incubations were carried out for 30 min (during which time SNAP-induced cGMP elevations remained relatively constant; Fig. S1A). In a first set of experiments, SNAP and immediately thereafter Na_2_S (or the vehicle controls) were added directly to the treatment medium at the concentrations indicated. In a second set of experiments, RFL-6 cells were pre-incubated with GYY4137 for 45 min, washed and then treated with SNAP. In a third set of experiments, stock solutions of nitrosopersulfide (SSNO^−^) were prepared by reacting 1 mM SNAP with 10 mM Na_2_S in 1 ml PBS or TRIS 100 mM pH 7.4. In a typical experiment, 10 µl of SNAP (100 mM/DMSO) and 100 µl Na_2_S (100 mM/Tris 100 µM pH 7.4) were added to an amber centrifugation tube containing 890 µl Tris 100 µM pH 7.4. After 10 min of incubation at RT, excess sulfide was removed either by precipitation with ZnCl_2_ (20 µl of 500 mM ZnCl_2_ to 1 ml yellow mixture; final concentration 10 mM), followed by a 1 min centrifugation at maximal speed, or by 10 min bubbling of the solution with nitrogen. Removal of sulfide was verified spectrophotometrically. The mix (2 µl, 20 µl, 200 µl) was then added to cell culture medium to reach a total volume of 1 ml (corresponding to a 1:500; 1:50; 1:5 dilution). In selected experiments, an sGC inhibitor (ODQ, 500 µM) or an NO scavenger (cPTIO, 500 µM) were added 5 min prior to the addition of sulfide and/or SNAP. The above dilution steps bring the theoretical maximal concentration of NO-containing bioactive material into the micromolar range. No signs of cell toxicity were observed following either of these treatments, as assessed by micro/macroscopic cell inspection and determination of protein content; the latter would have been reduced if a significant number of apoptotic/dead cells detached from the plates.

Cells were lysed in 0.1 M HCl at RT for 20 min, scraped, sonicated twice for 30 s at 4 °C, centrifuged at 30,000*g* for 10 min at 4 °C to remove cell debris, and the supernatant snap-frozen in liquid N_2_ and kept at −80 °C until analysis. Protein concentrations in the supernatant were determined by a modified Bradford’s protein assay (Roti^®^Nanoquant, Carl Roth GmbH+Co. KG, Karlsruhe, Germany) after pH equilibration with 200 mM TRIS pH 8. Intracellular cGMP levels were assessed by using DetectX^®^High Sensitivity Direct Cyclic GMP kit by Arbor Assay (Biotrend, Cologne, Germany) as per manufacturer’s instructions. Data were normalized for protein content and expressed as pmoles/ml/mg protein or as ratios compared to untreated cells. Changes in intracellular cGMP levels, normalized for protein content, were expressed as % of untreated control to further account for the variability in basal cGMP levels of untreated cells of different batches and passages (Fig. S1A, insert).

### Determination of intracellular sulfide levels

Intracellular sulfide levels were determined by flow cytometry following loading of RFL-6 cells with 10 µM WSP-1 for 30 min. After washing with 1 ml pre-warmed PBS, cells were detached by addition of 2 ml Accutase^®^ (PAA), and intracellular green fluorescence (λ_ex_ 488 nm, *λ*_em_ 519 nm) was assessed in a FACS-Verse flow cytometer (BD Bioscience, Heidelberg, Germany). Data were calculated as median fluorescence intensity (MFI) of loaded cells – background (ΔMFI) by plotting side-scatter (SSC) vs. fluorescence intensity in the FITC channel using FlowJo 10.0.6 (Tristar, Ashland, OR, USA).

### Measurement of phopshodiesterase activity

RFL-6 cells cultured for 10 or 18 passages, were scraped, lyzed in homogenization buffer (triethanolamine (TEA)/HCl 50 mM, NaCl 50 mM, EDTA 1 mM, DTT 2 mM, benzamidine 0.2 mM, phenylmethylsulfonyl fluoride 0.5 mM and 1 µM pepstatin A, pH 7.4, 4 °C), and cleared by centrifugation (800×g, 5 min, 4 °C). PDE activity in cell homogenates was measured by the conversion of [^32^P]cGMP or [^32^P]cAMP (synthesized from [α-^32^P]GTP or [^32^P]ATP using purified NO-sensitive guanylyl or adenylyl cyclase) to guanosine or adenosine and [^32^P]phosphate in the presence of alkaline phosphatase at 37 °C for 7 min. Reaction mixtures (0.1 ml) contained 0.05–10 µl of the homogenates (~5 µg protein), [^32^P]cGMP or [^32^P]cAMP (~2 kBq), various cGMP concentrations (0.1, 1, or 5 µM), 12 mM MgCl_2_, 3 mM dithiothreitol, 0.5 mg/ml bovine serum albumin, 2 or 4 U of alkaline phosphatase, and 50 mM TEA/HCl, pH 7.4. Reactions were stopped by adding 900 µl ice cold charcoal suspension (30% activated charcoal in 50 mM KH_2_PO_4_, pH 2.3). After pelleting the charcoal by centrifugation, [^32^P]phosphate in the supernatant was quantified by scintillation counting.

### Reaction of sulfide with nitrosothiols

The spectroscopic and kinetic behavior of the reaction between sulfide and CysNO, GSNO and SNAP was followed by UV–visible spectroscopy in a FLUOstar Omega (BMG Labtech, Offenburg, Germany). Stock solutions (100 mM) were diluted 1:100 in 1 M TRIS pH 7.5, transferred to a UV-transparent 96-well plate (200 µl/well). The in-built automatic injector was filled with 50 mM Na_2_S solution for in-well titration experiments. Spectra (200–800 nm) were acquired before and every 2 s for 100 cycles after injection of 1–20 µl aliquots of Na_2_S. Spectra were analyzed using Omega data analysis software (BMG Labtech). All other studies were carried out in 3 ml-volume quartz cuvettes, kept at either 25.0 or 37.0±0.02 °C with continuous stirring (t2 peltier-type cuvette holder with TC1 temperature controller, Quantum Northwest, Liberty Lake, WA, USA) using a Cary 60 UV/vis spectrophotometer and analyzed using WinUV software (Agilent Technologies, Wokingham, Berkshire, UK). No differences in spectral changes were observed whether SNAP was incubated with sulfide in the presence or absence of DTPA (100 µM), indicating that transition metal contamination of our buffers was negligible.

### Sulfide/disulfide nitrosation on solid phase

A stationary nitrosothiol (RSNO) column was prepared using batch nitrosation of the thiol-containing resin, Ekathiol (Sigma-Aldrich; Lot 115H1121) in a small beaker. Briefly, 0.4 g dry resin was suspended in and washed extensively with ultrapure water before reduction of resin-bound sulfhydryl groups (0.71 mmol thiol/g resin) with a 10-fold molar excess of dithiothreitol (in 0.01 M NaOH) for 5 min at RT; thereafter, the reductant was removed by 5 successive washes with ultrapure water. Free sulfhydryl groups were nitrosated by addition of acidified nitrite (NaNO_2_ in 1 M HCl; 2-fold molar excess), and allowed to react for 30 min at 4 °C in the dark. After a minimum of 5 washing steps with 10 ml ice-cold ultrapure water, the resin was equilibrated to pH 8.0 with 100 mM phosphate buffer and filled into two Pasteur pipettes with glass wool at the bottom. These mini-columns were washed 10 times with ice-cold phosphate buffer under dimmed lighting conditions. Sub-stoichiometric amounts of Na_2_S or Na_2_S_2_ in phosphate buffer were loaded onto the columns and eluted with ice-cold phosphate buffer; fractions were collected directly into 1 ml quartz cuvettes and UV–vis spectra recorded using an Agilent 8453 diode array spectrophotometer and ChemStation software (Agilent Technologies).

### Kinetics of NO release

In situ NO formation from SNAP (1 mM) with/without Na_2_S (0.1–10 mM) in the presence or absence of the metal chelator DTPA (100 µM) in 1 M Tris or 100 mM phosphate buffer, pH 7.4, was monitored by gas phase chemiluminescence (CLD 77am sp; Ecophysics, Dürnten, Switzerland) using a custom-designed, water jacketed glass reaction chamber (15 ml total volume) kept at 25±0.1 °C and continuously bubbled with nitrogen. 1 ml volumes of the respective SNAP/sulfide mixtures were premixed in Eppendorff vials, vortexed, and at the indicated time interval of incubation a 25 µl aliquot of the reaction mixture was transferred into the reaction chamber containing 15 ml buffer (dilution 1: 600) by means of a gas-tight syringe. NO concentration in the sample gas was recorded continuously and peak areas were integrated using an EPC-500/PowerChrom data processing system (eDAQ; Red Box Direct, Dublin, Ireland).

### Oxygen consumption measurements

Changes in dissolved oxygen concentration in incubation mixtures of SNAP and sodium sulfide (in 100 mM phosphate buffer pH 7.4, as above) were monitored polarographically using a dual channel Clark-type electrode system (Digital Model 20; Rank Brothers Ltd, Cambridge) and LabChart (ADInstruments, Oxoford, UK); incubation volume was 3 ml; solutions were maintained at a constant temperature of 37±0.5 °C using a circulating water bath. Reactions were typically started by the addition of SNAP to prewarmed sulfide-containing buffer solutions while continuously stirring, although the sequence of addition of sulfide or nitrosothiol did not have any effect on measured rates of oxygen consumption.

### Statistical analysis

Data are reported as means±SEM. ANOVA followed by an appropriate *post hoc* multiple comparison test (Tukey or Student's *T* test) was used to test for statistical significance.

## Results

### Sulfide modulates nitrosothiol bioactivity in a concentration-dependent manner

Nitrosothiols are known to activate and modulate sGC activity in vascular tissue and a wide variety of cellular preparations [32], [33], [34]. In this study, potential changes in nitrosothiol bioactivity by sulfide were investigated by analyzing SNAP-induced sGC activation in RFL-6 cells, a convenient NO reporter system lacking an active NO synthase and expressing low levels of PDE5 [31]. Total PDE activity in RFL-6 cells was found to be greater for cAMP than cGMP (295±15 vs. 129±14 pmol/mg/min, p10; *n*=2) and decreased on further passaging (111±8 vs 81±3 pmol/mg/min, p17; *n*=2). To minimize the effects of cell passage-dependent and sulfide-induced variations in PDE activity on intracellular cGMP levels, experiments were carried out in the presence of IBMX.

Using this system we found that sulfide modulates the NO bioactivity of SNAP to varying degrees, depending on the relative concentration ratio of sulfide over SNAP. Low sulfide concentrations (1–10 µM) inhibited sGC stimulation by 100 µM SNAP (Fig. 1A), whereas cGMP levels with equimolar concentrations of sulfide and SNAP were not different from those of SNAP alone (Fig. 1A). Similar results were obtained when intracellular sulfide levels were elevated by pre-incubating cells with the sulfide donor GYY4137. Pre-incubation with 10–100 µM GYY4137 increased intracellular fluorescence of the sulfide-specific probe WSP-1 (Fig. S1B) and inhibited SNAP-induced cGMP increases (Fig. 1B) in a concentration-dependent fashion. Unexpectedly, we found that high concentrations of sulfide (100 µM Na_2_S or 1 mM GYY4137) increased cGMP even in the absence of SNAP (Fig. 1A and C). These sulfide-mediated cGMP increases were inhibited by either cPTIO or ODQ, but unaffected by the presence/absence of IBMX (Fig. 1C and D), indicating that the cGMP changes observed were NO/sGC-dependent but independent of PDE activity. Whether or not preformed NO-storage pools may account for this effect of sulfide warrants further investigation. SNAP-induced sGC activation was abolished by treating cells with the sGC inhibitor ODQ or the NO scavenger cPTIO (see Fig. 1D), indicating that effects were dependent on the release of NO with consecutive activation of sGC. Taken together, these results show that low concentrations of sulfide attenuate SNAP bioactivity whereas sGC stimulation by SNAP is fully restored at equimolar sulfide levels.

### The reaction of sulfide with nitrosothiols leads to formation of more than one NO-releasing species

**Fig. 2:**
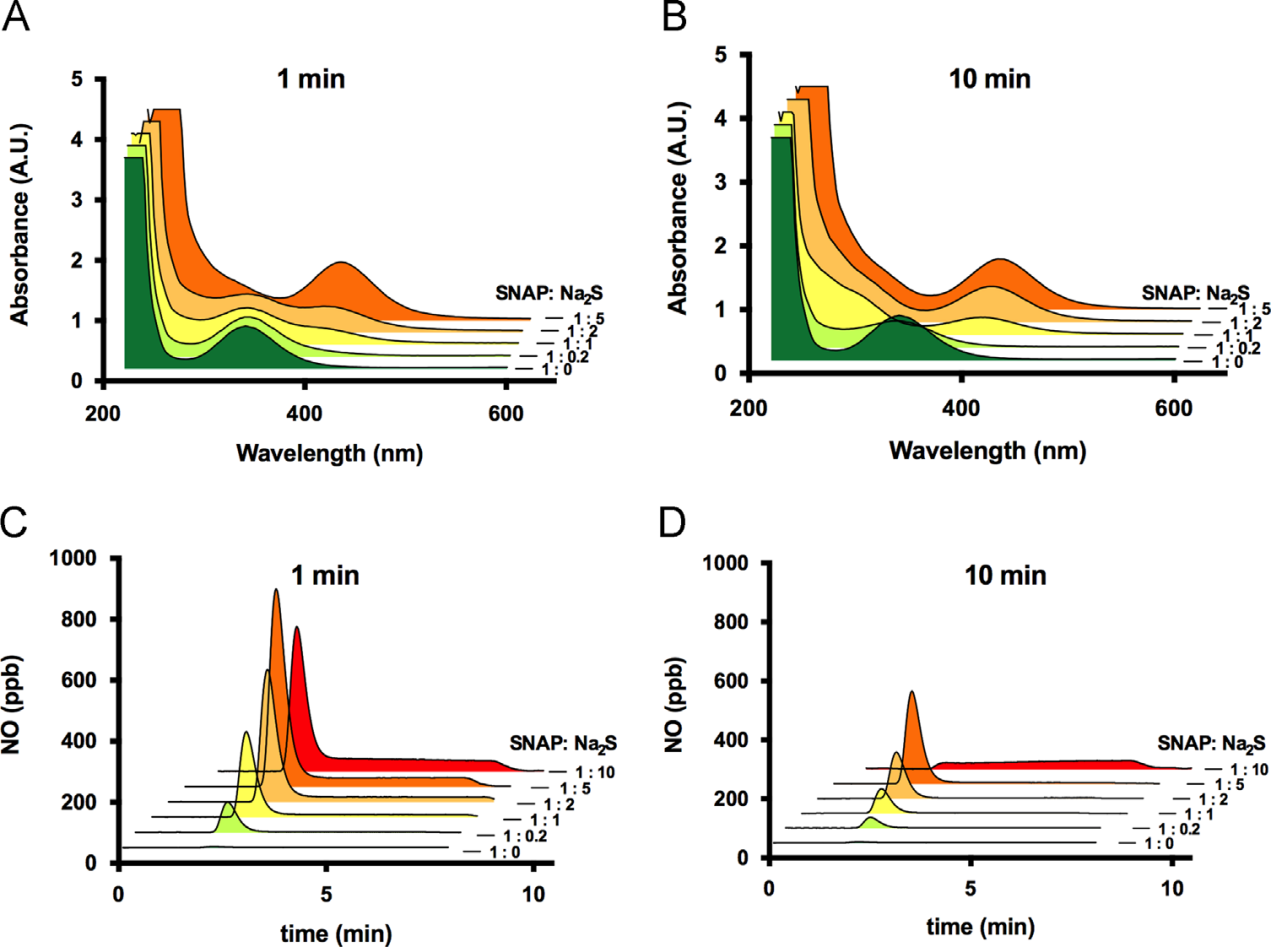
The reaction between sulfide and nitrosothiols leads to formation of NO-releasing species. (A,B) UV–visible spectroscopic analyses of the reaction between SNAP and hydrosulfide at pH 7.4. Varying SNAP/Na_2_S concentration ratios lead to formation of different products as assessed after 1 min (A) and 10 min of incubation (B); with excess sulfide accumulation of a ‘yellow compound’ (*λ*_max_ 412 nm) was observed. (C,D) Profiles of NO release from SNAP/Na_2_S mixtures of different concentration ratios after 1 min (C) or 10 min of incubation (D). (For interpretation of the references to color in this figure legend, the reader is referred to the web version of this article.)

**Fig. 3:**
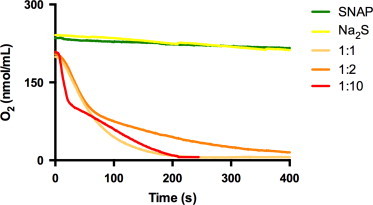
Oxygen consumption by the reaction of SNAP with sulfide. Changes in dissolved oxygen (O_2_) concentration in air-saturated phosphate buffer pH 7.4 by 1 mM SNAP (green tracing) or 2 mM Na_2_S alone (yellow), as compared to mixtures of 1 mM SNAP and different concentration of sulfide (1, 2, 10 mM, final concentrations). Representative tracings of 3–4 separate runs at each condition yielding qualitatively identical results. (For interpretation of the references to color in this figure legend, the reader is referred to the web version of this article.)

In order to characterize the chemical biology of interaction between SNAP and sulfide, we monitored the reaction using time-resolved UV/vis spectrometry and gas phase chemiluminescence (Fig. 2). SNAP decomposition (decrease in *λ*_max_ at 340 nm) was accompanied by absorbance increases in the region of 250–300 nm and 390–430 nm with a peak at *λ*_max_ 412 nm (Fig. 2A and B), and – under some conditions – transiently around 320 nm (Fig. 2, Fig. 3A), indicative of the formation of multiple reaction products/intermediates. Nature, yield, rate of formation, and stability of these products were strictly dependent on the concentration ratio of the reactants (see Fig. 2A and B). Similar spectral changes, albeit with different kinetics, were observed when sulfide was allowed to react with two other nitrosothiols, CysNO and GSNO (Figs. S2A, S2B). To investigate the potential of reaction products to generate NO after complete decomposition of the starting material, aliquots of the reaction mixture of SNAP and sulfide were subjected to chemiluminescence analyses at different time points of co-incubation.

Transition metals in aqueous buffers can trigger nitrosothiol decomposition [35]; in our hands, SNAP alone produced a very small NO signal when experiments were carried out in the presence of the metal chelator DTPA (100 µM; Fig. 2C and D). However, peak NO release was markedly enhanced when SNAP was pre-incubated for 1 min with sulfide prior to injection into the reaction chamber (Fig. 2C). This enhancement of rate of NO formation was apparent at sub-stoichiometric concentrations of sulfide and became progressively more prominent as sulfide concentrations increased (1:1, 1:2, 1:5 and 1:10), seemingly matching the extent of formation of the 412 nm peak (Fig. 2A). A biphasic NO release profile was observed at higher sulfide concentrations, with a sharp initial peak followed by a more sustained lower level of NO production. Injection of aliquots of the reaction mixture that had been pre-incubated for 10 min revealed a decreased NO releasing capacity (Fig. 2D), indicative of ongoing decomposition of NO-generating entities. The long-lasting NO releasing component was observed only at higher sulfide concentration ratios. Since SNAP itself produces just traces of NO under these conditions (and the absorbance feature at 340 nm rapidly disappears on incubation with excess sulfide), the NO formation we detected could not have originated from SNAP itself; thus other products capable of releasing NO must have been formed in the course of the reaction.

### Nitrosothiol/sulfide reactions are accompanied by the consumption of oxygen

Solutions of either NO or sulfide are known to slowly react with oxygen (autoxidation) [2], [8]. In order to investigate whether the chemical interaction of sulfide with nitrosothiols affects the concentration of dissolved oxygen beyond that expected by the sum of either reactant alone we monitored oxygen consumption in SNAP/sulfide incubation mixtures. As depicted in Fig. 3, oxygen consumption rates accelerated dramatically on coincubation of SNAP and sulfide, exceeding that of either reaction partner by orders of magnitude. Although higher molar ratios of sulfide over SNAP tended to hasten oxygen consumption compared to equimolar levels no simple concentration dependence was apparent, with well-behaved second/pseudo first order rates adopting a biphasic kinetic profile whenever sulfide was in excess over SNAP. Such changes are difficult to rationalize by a simple enhancement of NO production alone but consistent with the formation of more reactive radical intermediates.

### A ‘yellow compound’ is produced by reaction of nitrosothiols with excess sulfide and assigned to be nitrosopersulfide, SSNO^−^

**Fig. 4:**
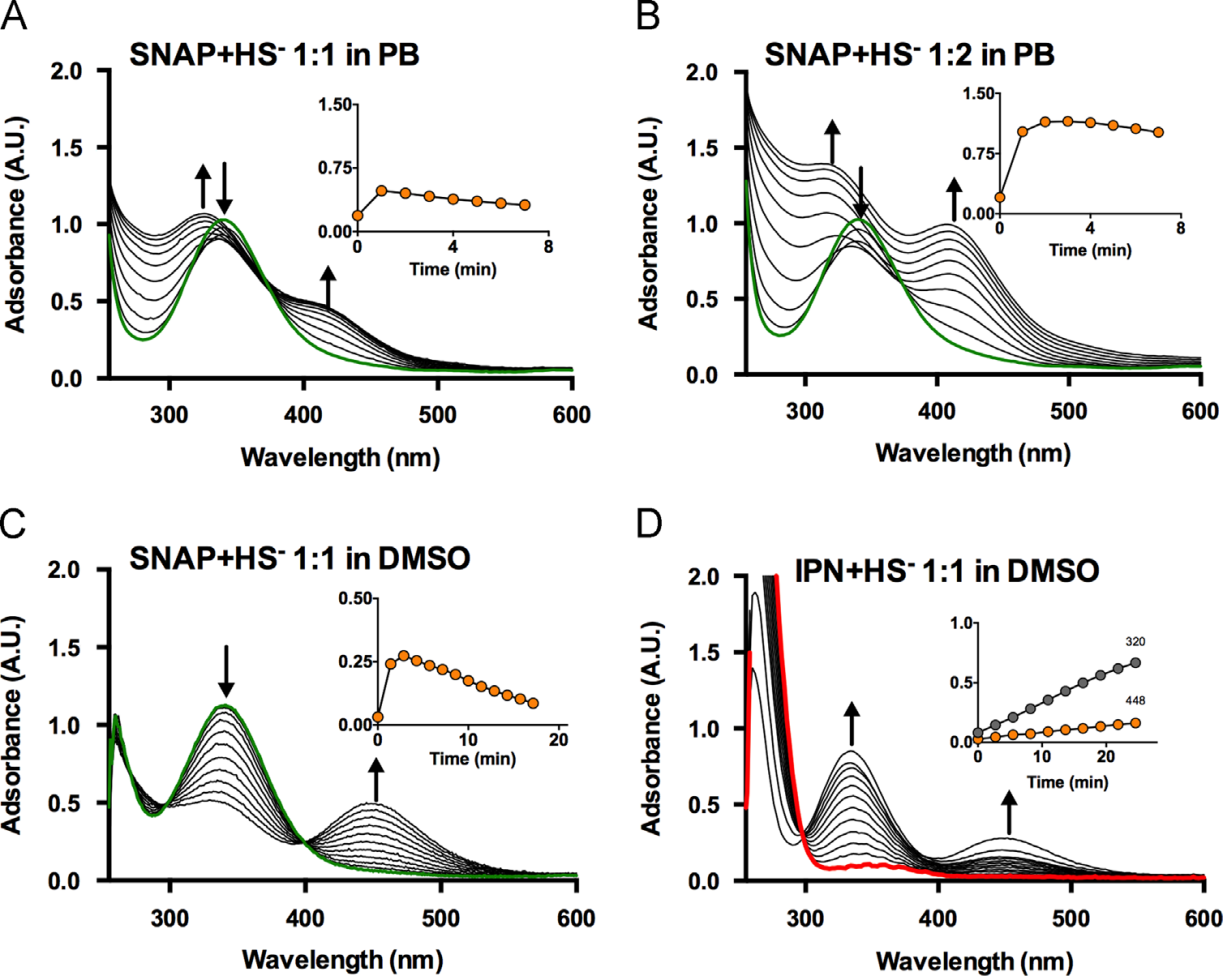
Formation of SNO^−^ and SSNO^−^ during the reaction of SNAP or isopentyl nitrite (IPN) with hydrosulfide (HS^−^) under aqueous and non-aqueous conditions. (A,B) Reaction of Na_2_S and SNAP in phosphate buffer (PB) at pH 7.4. Green line, starting spectrum of SNAP. Attributions: *λ*_max_ 320 nm=SNO^−^; *λ*_max_ 412 nm=SSNO^−^ (C) Reaction of Na_2_S and SNAP in DMSO. Spectra taken in the first 60 s after the beginning of the reaction are shown. Attributions *λ*_max_ 448 nm=SSNO^−^ (D) Reaction of isopentylnitrite (IPN) with Na_2_S in DMSO. Red line, starting spectrum of IPN. Attributions: *λ*_max_ 325 nm=ONS^−^; *λ*_max_ 448 nm=SSNO^−^. Time interval for spectra: 0–60 s. Cycle time 0.1 s. Insets in (A–D) are depicting the time course of absorbance changes at 412 nm (A,B) or 448 nm (C,D).

A major product of the reaction of SNAP (and other nitrosothiols, as demonstrated for CysNO and GSNO in Figs. S2A, S2B) with excess sulfide consists of a ‘yellow compound’ with a strong absorbance feature at 412 nm (Fig. 2A and B; Fig. 4A and B). Earlier work by Seel et al. [36] and Munro et al. [37] had described the formation of a similar absorbance feature with a maximum at 409–410 nm in the course of the reaction between sodium sulfide and NO or nitrosothiols, respectively; those experiments had been carried out in unbuffered strongly alkaline solutions, and both groups had attributed this peak to the formation of the stable yellow-colored nitrosodisulfide (perthionitrite) anion, SSNO^−^. Our own experiments confirmed that the absorption maximum of the ‘yellow compound’ shows a small bathochromic shift on further alkalinization. When the reaction of SNAP and sulfide was carried out in polar organic solvents such as DMSO (Fig. 4C) or DMF (not shown) the absorbance maximum shifts to 448 nm, again consistent with observations by Seel et al. [38]. Under these conditions, another prominent absorbance feature with a maximum at 330 nm occurs (Fig. 4C). According to Seel et al. [38], this peak is indicative of the formation of thionitrite (SNO^−^). The formation of either species was observed also when sulfide was reacted with the classical nitrosating agent, isopentyl nitrite (IPN), instead of SNAP; in this case, SSNO^−^ formation was less pronounced and clearly preceded by the formation of SNO^−^ (Fig. 4D).

**Fig. 5:**
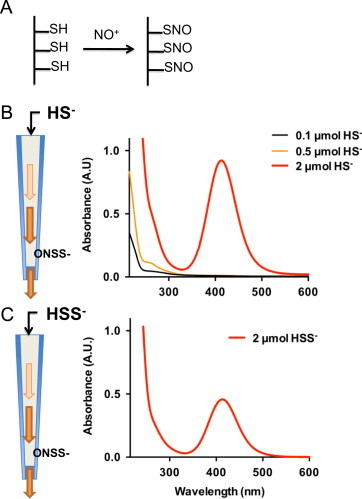
Formation of SSNO^−^ from the reaction between sulfide and immobilized S-nitroso moieties. (A) S-nitrosation by acidified nitrite of resin-bound sulfhydryl (–SH) groups (Ekathiol^®^) to form an immobilized nitrosothiol. (B,C) A solution of either Na_2_S (B) or Na_2_S_2_ (C) is passed over the column and reacts with immobilized S-nitrosothiols to form SSNO^−^ (*λ*_max_ 412 nm; high sulfide concentration) and polysulfides (<280 nm; lower sulfide concentrations). Depicted results are representative of 4 independent experiments with different batches of nitrosated thiol-containing resin and various absolute amounts of sulfide (0.015–5 µmoles on column).

The reaction between sulfide and nitrosothiols was also carried out on solid phase by passing aqueous solutions of sulfide (0.1, 0.5, 2 µmoles in 100 µl of 100 mM phosphate buffer, pH 8) over a resin containing immobilized S-nitrosothiol moieties serving as a stationary nitrosonium (NO+) donor (Fig. 5). The higher concentration of sulfide produced a faint yellow band in the upper part of the packed resin that turned intensely yellow/orange on migrating down the column; this was accompanied by the formation of gas bubbles. The lower and medium concentrations of sulfide produced a step-like absorbance feature in the UV range, indicative of the formation of polysulfides. The absorption spectrum of the yellow eluate collected from the high sulfide fraction revealed a broad symmetrical peak between 320 and 500 nm (*λ*_max_ 412 nm). An identically colored solution with a peak absorbance at 412 nm was obtained when sodium disulfide (Na_2_S_2_) instead of Na_2_S solutions were passed over the column.

### NO release from nitrosopersulfide accounts for sustained sGC activation by SNAP

**Fig. 6:**
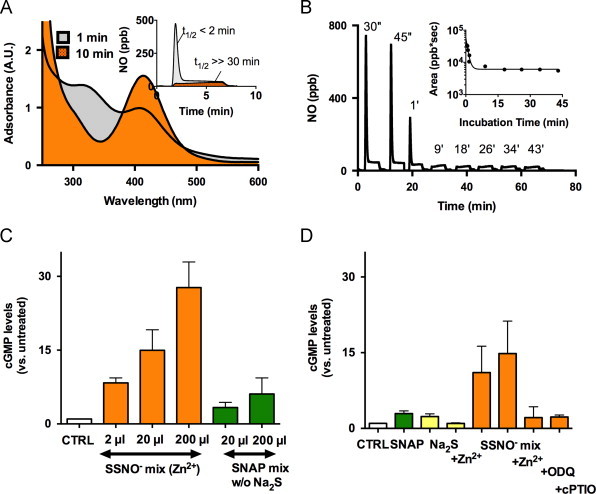
Decomposition of SSNO^−^ releases NO and activates of sGC. (A) UV–visible spectra (main panel) and chemiluminescent profile of NO release (inset) of a SNAP/Na_2_S mixtures (1:10) at pH 7.4 following 1 min (gray) or 10 min (orange) of incubation at RT in 1 M TRIS buffer, pH 7.4. (B) Original chemiluminescence tracing of NO release over time of a SNAP/Na_2_S mixture (1:10) incubated for the indicated time points. Inset: changes in total NO release (area under the curve) with increasing time of preincubation. (C) Dose-dependent sGC activation by the SNAP/Na_2_S mixture (1:10) incubated for 10 min (SSNO^−^ mix) compared to the same volumes of a control mix prepared with SNAP only (SNAP mix). (D) NO and sGC dependent cGMP increase in RFL-6 cells after incubation with 20 µl of the SSNO^−^ mix compared to the effects of SNAP and sulfide (Na_2_S) alone. “+Zn^2+^” denotes removal of excess sulfide by zinc precipitation; indicated volumes were added directly to the cell culture medium to reach a total volume of 1 ml. (For interpretation of the references to color in this figure legend, the reader is referred to the web version of this article.)

The peak at *λ*_max_ 412 nm assigned to SSNO^−^ was found to be rather stable at physiological pH (t_1/2_>>30 min at RT; Fig. 6A, inset). Given this anionic compound is formally a nitrosothiol we were surprised to see that its lifetime was seemingly unaffected by the presence of excess sulfide and/or addition of other reduced thiols (tested for glutathione and cysteine, both at 1 mM; Fig. S3). Addition of a strong base (NaOH) did not affect the stability of this substance either (Fig. S3), but acidification led to immediate disappearance of the yellow color with its absorbance feature at 412 nm, followed by formation of a colloidal suspension indicative of sulfur extrusion (not shown). By contrast, decomposition of the ‘yellow compound’ at physiological pH leads to formation of polysulfides (Fig. S4), which are susceptible to decomposition following reaction with dithiothreitol (DTT, 1 mM); no precipitation of sulfur was observed in the presence of excess sulfide.

Evidence for NO production during SSNO^−^ decomposition was obtained using SNAP/Na_2_S (1 mM/10 mM) mixtures that were pre-incubated for at least 10 min, and strongly diluted prior to testing by gas phase chemiluminescence (Fig. 6A and B; dilution 1:600) and sGC activation in RFL-6 cells (Fig. 6C and D; 1:5 to 1:500 dilution). Repeated injections of small 25 µl aliquots of the reaction mixture allowed the determination of an apparent ‘half-life of the NO releasing activity’ of the reaction products; as observed with the 412 nm peak (Fig. S3), the decomposition of the SSNO^−^ specific portion of the NO peak occurred over a similar timescale (Fig. 6A and B). Parallel investigations of the SNAP/sulfide reaction mixture revealed a concentration-dependent increase in cellular cGMP levels in RFL-6 cells (Fig. 6C and D). Importantly, sGC activation by SSNO^−^ was considerably more pronounced than that seen with SNAP (or sulfide) alone (Fig. 6C,D), blocked by addition of the NO scavenger cPTIO and the sGC inhibitor ODQ (Fig. 6D). Removing excess sulfide by zinc precipitation immediately before addition of the preincubated SNAP/sulfide mixture to the cells had either no effect on or slightly enhanced sGC activation by SSNO^−^ (Fig. 6D).

## Discussion

We here demonstrate that 1. sGC stimulation by nitrosothiols is modulated by variation in extracellular and intracellular sulfide levels; 2. chemical reaction of sulfide with nitrosothiols leads to distinct products, depending on the concentration ratio of the reactants; 3. with excess sulfide over nitrosothiols this reaction leads to formation of a stable ‘yellow compound’ which we assign to be nitrosopersulfide (SSNO^−^); and 4. SSNO^−^ decomposition generates enhanced NO bioactivity over that of SNAP alone. 5. Given that sulfide concentrations likely exceed those of nitrosothiols in most biological compartments, our data suggest that SSNO^−^ is formed whenever NO and H_2_S are co-generated and interact, thus contributing to the biological activity of both “hydrogen sulfide” and nitrosothiols. Considering the apparent stabilities of thionitrite (SNO^−^) and nitrosopersulfide in the presence of reduced thiols (incl hydrosulfide), the latter rather than the former is likely to account for the sustained NO bioactivity of nitrosothiols. Taken together, our studies imply that SSNO^−^ is a new signaling entity with the potential to generate both NO and polysulfides.

### Chemistry of formation and decomposition of nitrosopersulfide

One of the most interesting aspects of our present studies relates to the observation that a ‘yellow compound’ is formed and accumulates whenever sulfide is in excess over nitrosothiols. On the basis of its spectral characteristics, and in agreement with work by Seel and Wagner [36], [38] as well as Munro and Williams [37], we assign this product to be nitrosopersulfide (SSNO^−^), a hybrid species containing a sulfane sulfur and a nitrosonium moiety attached to opposite ends of a sulfur atom. It formally belongs to the group of polysulfides (S_*x*_^2−^) and is its smallest NO-containing representative. Such a compound would seem to be of particular interest in the context of the cross-talk between the NO and the H_2_S signaling pathways, an area of research that has attracted much interest lately, due to its propensity to generate both, NO and polysulfides/sulfane sulfur; the latter are becoming increasingly recognized as potent bioactive sulfide metabolites [39], [40].

In the absence of transition metals or light, the homolytic decomposition of nitrosothiols (RSNO) to yield NO and a thiyl radical (Eq. 1) is a fairly slow process because the S–N bond is relatively strong [41].

(1) $$\mathrm{RSNO}\to {\mathrm{RS}}^{•}{+}^{•}\mathrm{NO}$$

However, nitrosothiols are unstable in the presence of reduced thiols. The expected reaction between a nitrosothiol and hydrosulfide, the smallest thiol and one of the strongest nucleophiles of physiological relevance, is transnitrosation to give thionitrous acid, a well-established if unstable species [42](Eq. 2).

(2) $$\mathrm{RSNO}+{\mathrm{HS}}^{-}\to {\mathrm{RS}}^{-}+\mathrm{HSNO}$$

Transnitrosation is a rapid reaction, and all of the HS^−^ should be converted into HSNO. Assuming a pKa for the latter similar to that of nitrous acid (HONO), at physiological pH this species is expected to exist largely in the form of the corresponding anion, thionitrite (SNO^−^); in alkaline solution, thionitrite has an absorption maximum near 320 nm [43], [44], which corresponds to one of the transient spectral features we observed in the present study (see Fig. 2, Fig. 3).

Nitrosothiols act as vasodilators because of their ability to donate NO to sGC. HSNO might be expected do the same but, because of the mobile hydrogen in the molecule, it can undergo a number of other reactions, such as facile isomerization to three other species with the same formula (HNSO, HOSN, HSNO, HONS and their corresponding anions, respectively; (Eq. 3) [25]).

(3) HNSO⇌HOSN⇌HSNO⇌HONS

Although the decomposition products of these species have not been investigated in great detail, they appear to be largely gaseous and/or form polymeric material [45]. Thus, HSNO will not deliver to sGC a stoichiometric amount of NO, as does RSNO. As a result, formation of HSNO/SNO^−^ results in reduced stimulation of sGC.

In the presence of higher concentrations of HS^−^ the situation is rather different as another reaction (nucleophilic attack of HS^−^) becomes dominant.

(4) $$\mathrm{HSNO}+{\mathrm{HS}}^{-}\to \mathrm{HSSH}+{\mathrm{NO}}^{-}$$

(5) $$\mathrm{HSSH}⇌H^{+}+{\mathrm{HSS}}^{-}$$

This is a second order reaction (rate=*k*[HSNO][HS^−^]) and so, while negligible at low concentrations of HS^−^, at higher hydrosulfide concentrations it dominates. The persulfide (hydrodisulfide anion; HSS^−^) formed is also a strong nucleophile; it can undergo transnitrosation with RSNO in a fashion similar to HS^−^.

(6) $$\mathrm{RSNO}+{\mathrm{HSS}}^{-}\to \mathrm{RSH}+{\mathrm{SSNO}}^{-}$$

The resulting nitroso compound, nitrosopersulfide (perthionitrite; SSNO^−^) appears, from our work and that of Seel et al. [36], [43], to be considerably more stable than HSNO and, therefore, a better vasodilator. The formation of sulfur chains, admittedly a chain of only two in this instance, is so common in sulfur chemistry that to find evidence for the long-term presence of HSNO would be surprising. Longer sulfur chains are common at higher concentrations, and elemental sulfur exists, of course, as S_8_. It is not unreasonable to postulate that, because of its enhanced stability, HSSNO/SSNO^−^ is better able to provide sGC with NO. At the same time it is more mobile than RSNO and therefore a more versatile vasodilator. Some of the NO moiety is lost as nitroxyl anion, NO^−^ (Eq. 4), but vascular tissue can readily convert this to NO [46]. Thus, high concentrations of HS^−^ enhance the vasodilator activity of nitrosothiols.

Alternative routes to nitrosopersulfide formation may exists when thiol disulfides (RSSR’) are present; in this case, alkyl persulfides (RSS^−^) may be formed [47], which may serve as a source of HSS^−^ (Eqs. (6), (7)).

(7) RSSR'+HS−→RSSH+R'S−

(8) $$\mathrm{RSSH}+{\mathrm{HS}}^{-}\to {\mathrm{RS}}^{-}+\mathrm{HSSH}$$

Nitrosopersulfide may also be formed via a radical pathway that starts with the homolytic cleavage of HSNO to generate sulfanyl (hydrosulfide radical, HS^•^), which is extremely reactive and immediately reacts with excess thiolate (in this case, HS^−^) to form the hydrodisulfide radical (HSS^•−^).

(9) $$\mathrm{HSNO}\to {\mathrm{HS}}^{•}{+}^{•}\mathrm{NO}$$

(10) $${\mathrm{HS}}^{•}+{\mathrm{SH}}^{-}\to {\mathrm{HSS}}^{\cdot -}$$

This species then either reacts further with NO (to form nitrosopersulfide; Eq. 11) or oxygen (to form superoxide; Eq. 12).

(11) $${\mathrm{HSS}}^{•-}{+}^{•}\mathrm{NO}\to \mathrm{HSSNO}⇌{\mathrm{SSNO}}^{-}+H^{+}$$

(12) $${\mathrm{HSS}}^{•-}+O_{2}\to {\mathrm{HSS}}^{-}+{O_{2}}^{•-}$$

Thus, SSNO^−^ might form via several different avenues [37]. Formation via HSNO seems a plausible route, since isopentyl nitrite (a prototypical nitrosating agent characterized by an alkyl nitrite (R-ONO) grouping) was demonstrated here to undergo a similar reaction in that it first leads to SNO^−^ generation, with SSNO^−^ formation becoming apparent only much later (Fig. 4D). Those experiments were carried out in DMSO (as shown here) and DMF. The advantage of using these non-aqueous ‘electron pair donor’ solvents is that under these conditions, anions exist essentially in their “naked” form (unlike in water where solvation is via formation of hydrogen bonds), resulting in an enhancement of their nucleophilicity. In the case of SSNO^−^, a large bathochromic shift from 412 nm (in aqueous solution) to 450 nm is observed; for SNO^−^ a lesser shift was apparent but a marked enhancement of molar UV-absorbance was seen. In DMSO/DMF one can also observe the vibrational fine structure of the R-ONO absorbance feature superimposed onto the broad nitrite peak while allowing for convenient monitoring of the formation of SNO^−^ and SSNO^−^ at the same time. The same characteristic changes take place when the reaction of nitrosothiols with HS^−^ is carried out in those solvents, as we here demonstrate for SNAP and sulfide (compare Fig. 4A with 4C). Interestingly, the same final reaction product was formed when aqueous solutions of either Na_2_S or Na_2_S_2_ were passed over a stationary nitrosothiol column (Fig. 5). Thus, the above routes are neither mutually exclusive, nor may they represent the only pathways through which SSNO^−^ can be formed. Conceivably, multiple reaction pathways may occur in parallel, which would explain the complexity of spectral interconversions taking place (as becomes apparent when one analyzes sequential spectra of reaction mixtures and their absorbance-time records).

The products of SSNO^−^ decomposition are NO and polysulfides as evidenced in the present study by the measurement of gas phase chemiluminescence and sGC activation (Fig. 6) and the spectral step-like absorbance features in the UV range characteristic of polysulfides (Fig. 2, Fig. 4, Fig. 5 and S4). As expected, the potential of SNAP/HS^−^ mixtures to release NO correlated inversely with their peak absorbance at 412 nm, and as SSNO^−^ decomposes, polysulfide absorbance at 290–300 nm increases (Fig. S4).

(13) $${\mathrm{SSNO}}^{-}\to {\mathrm{SS}}^{•-}+{\mathrm{NO}}^{•}$$

(14) $${\mathrm{SS}}^{•-}\to {S_{4}}^{2-}\to \to ({S_{x}}^{2-})\overset{H_{2}O}{\to }{S_{\left(x-1\right)}}^{2-}+H_{2}S$$

Assuming homolytic cleavage of SSNO^−^, the most likely initial product formed in addition to NO is the disulfide radical, SS^•−^ (Eq. 13). Dimerization and reaction with excess thiolate may give rise to formation of higher polysulfides (${S_{x}}^{2-}$/HS_*x*_^−^). In aqueous solutions at pH 7.4, this is followed by hydrolysis and disproportionation reactions, yielding polysulfides of varying chain lengths in addition to free H_2_S [48] (Eq. 14).

What emerges from the above is that the reaction between nitrosothiols and sulfide is all but straightforward; this had already been observed by others before. Although carried out in unbuffered alkaline solutions rather than at physiological pH (and thus not directly comparable to the present observations at physiological pH), Munro et al. reported that the absorbance changes they observed at low sulfide concentrations were “not easily interpreted, and too complicated for a simple kinetic analysis,” possibly due to “several rapid processes … occurring” [37]. Indeed, the complexity of the UV/vis spectral changes seen during the reaction of nitrosothiols with sulfide at different concentration ratios demonstrate that the stability and nature of the intermediates formed strongly depend on the relative concentration ratio of the reactants, with thionitrite, nitrosopersulfide and NO likely not the only products formed in this reaction.

What exactly accounts for the scavenging of NO – as indicated by the inhibition of SNAP-induced cGMP production in RFL-6 cells – is unclear at present. Similar functional results have been reported by Moore and coworkers using other NO-donors [10], [21] but – to the best of our knowledge – this is the first time that an inhibition of NO bioactivity has been demonstrated for nitrosothiols. While the isomerization tendency of HSNO to form other products with lower NO generating potential likely plays a role, it is possible that additional NO-scavenging effects by reactive sulfur-oxy intermediates such as sulfite [49] or sulfur radical species are involved; the latter could either produce reactive oxygen species that scavenge NO by direct chemical reaction or act as temporary “NO sink” by forming S-nitroso species (with the potential to release NO further downstream, as demonstrated here for SSNO^−^). Attempts to mechanistically investigate the possible involvement of radical intermediates by using spin traps i.e. DMPO would be of little help in this context as they will also trap NO and thus by definition lower NO bioavailability. Yet, if thiyl radical chemistry was indeed involved the nitrosothiol/HS^−^ interaction should also be accompanied by a reaction with “mother nature’s spin trap”, molecular oxygen. In fact, our own preliminary investigations (Fig. 3) confirm that dissolved oxygen is consumed upon reaction of SNAP with sulfide at rates exceeding those of either NO and sulfide autoxidation by far. This observation is interesting not only from a chemical perspective, but also because mitochondrial sulfide oxidation has been linked to physiological oxygen sensing [50]. The reaction of nitrosothiols with sulfide would seem to add an interesting “twist” to that story. Thus, both the oxygen dependence of the reaction and the mechanism accounting for the drop in NO bioavailability at lower sulfide concentrations would seem to merit further investigation.

### Effects of sulfide on nitrosothiols bioactivity – is it all in the chemistry?

A series of recent investigations suggests that much of the H_2_S/NO cross-talk in the vasculature occurs via modulation of cGMP breakdown following inhibition of PDE activity [28], [51], [52]. Consistent with this notion, the PDE5 inhibitor sildenafil has been shown to attenuate the vasorelaxant effects of NaHS in rat aorta [51]. In rat smooth muscle cells, treatment with IBMX led to an increase in cGMP whereas sulfide itself had little effect [28]. While sulfide was unable to directly activate isolated sGC or modify sGC stimlation by the NO donor DEA/NO [52], low concentrations of sulfide were found to inhibit cellular PDE preparations [28] and recombinant PDE5 [51]. However, these results are not without controversy as others, using the endothelial cell line RF/6A, failed to demonstrate a cGMP increase by sulfide [53].

We find that sulfide-mediated cGMP elevations in RFL-6 cells were inhibited by ODQ and unaffected by the presence of IBMX indicating that these effects are dependent on sGC activity, i.e. the formation of cGMP rather than alterations in outward transport or enzymatic breakdown. Consistent with earlier reports [54], IBMX alone did not affect basal cGMP levels in RFL-6 cells, presumably as these cells do not have an active NO-synthase and express only low levels of PDE5 [29]. Accordingly, we did not observe any difference in SNAP-induced cGMP elevations from baseline in the presence or absence of IBMX; moreover, the cGMP-specific PDE activity of these cells was lower than the corresponding cAMP-related activity. Interestingly, the NO scavenger cPTIO significantly attenuated sGC activation by higher sulfide concentrations but had no effect on basal levels. This action profile is consistent with the notion that sulfide may contribute to sGC activation following direct chemical interaction with NO stores, although this possibility has not been experimentally verified. Taken together, the effects of sulfide on intracellular cGMP levels appear to be dependent on the interplay between cGMP production by sGC, cyclic nucleotide breakdown and possibly clearance pathways (e.g., alterations in cyclic nucleotide transport across membrane). If true, this might explain why responses to sulfide differ between cell type and vascular beds studied.

### Physiological relevance of the sulfide/nitrosothiol interaction

The potential of hydrosulfide (and by inference, H_2_S) to either attenuate or potentiate NO bioactivity may be of relevance to the regulation of vascular tone. From studies in rodent tissue we know that nitrosothiols, an important storage form of NO [55], [56], are particularly abundant in the vasculature [56]; the same seems to hold true for sulfide-related metabolites (at least in the aorta [57]), and both, endothelial and smooth muscle cells have been shown to express the enzymatic machinery capable of generating H_2_S [19], [58], [59]. Sulfide may therefore contribute to the fine-tuning of NO bioactivity in the vasculature, either via modulation of intracellular cGMP concentrations following inhibition of PDE activity [28], [51], [52] and/or, as demonstrated in the current study, by chemically reacting with nitrosothiols. Besides the transient formation of SNO^−^, the two major bioactive intermediates produced in the course of the latter reaction appear to include nitrosopersulfide and polysulfides. The concentrations used in the present proof-of-principle studies are clearly well outside the physiologically relevant range. Nevertheless, the effects of sulfide on SNAP-induced cGMP accumulation in RFL-6 cells, as shown in Fig. 1, suggest this interaction can occur also within cells; at present, this is mere speculation awaiting experimental confirmation by NMR and/or mass spectrometry based analytical techniques that allow unequivocal identification of SSNO^−^ formation in cells and tissues. However, since the outcome of this chemical cross-talk is strongly dependent on the relative concentration ratio of the reactants and linked to oxygen availability in several ways, in vivo experiments will ultimately be required to shed further light on the biological relevance of this interaction. The situation may be considerably more complex under pathophysiological circumstances as the kinetics of expression of the different enzymes required to sustain an enhanced production of both H_2_S and NO likely diverges over the time-course of induction in different tissue compartments, giving rise to an interesting dynamic in what already looks like a complex interaction scenario under controlled conditions. The present observations may also help explain some of the conflicting results regarding the NO/sulfide interaction obtained by different investigators.

## Conclusion

In summary, we have demonstrated that the chemical reaction of nitrosothiols with sulfide leads to modulation of their NO-related bioactivity in a concentration-dependent fashion, giving rise to inhibition at low and potentiation at higher sulfide concentrations. Spectral evidence demonstrates that under conditions of excess sulfide SSNO^−^ is formed, which release NO at higher rate and thus activates sGC more effectively than the starting nitrosothiol itself. While further detailed study is warranted to identify the chemical nature and biological activity of other reaction products involved, the chemical and biological properties of SSNO^−^ suggest that nitrosopersulfide represents a new signaling entity at the cross-roads between sulfide and NO/polysulfide signaling.

## Footnotes

Appendix A Supplementary data associated with this article can be found in the online version at doi:10.1016/j.redox.2013.12.031.
