# Supplementary material for: Nitrosopersulfide (SSNO−) accounts for sustained NO bioactivity of S-nitrosothiols following reaction with sulfide
Source: Redox Biol. 2014 Jan 11;2:234–44. doi: 10.1016/j.redox.2013.12.031 (PMC3909780; doi:10.1016/j.redox.2013.12.031)
Supplement: Supplementary file 1 — Supplementary data [file mmc1.doc]

# Supplementary Figures:

**Figure S1:**

**
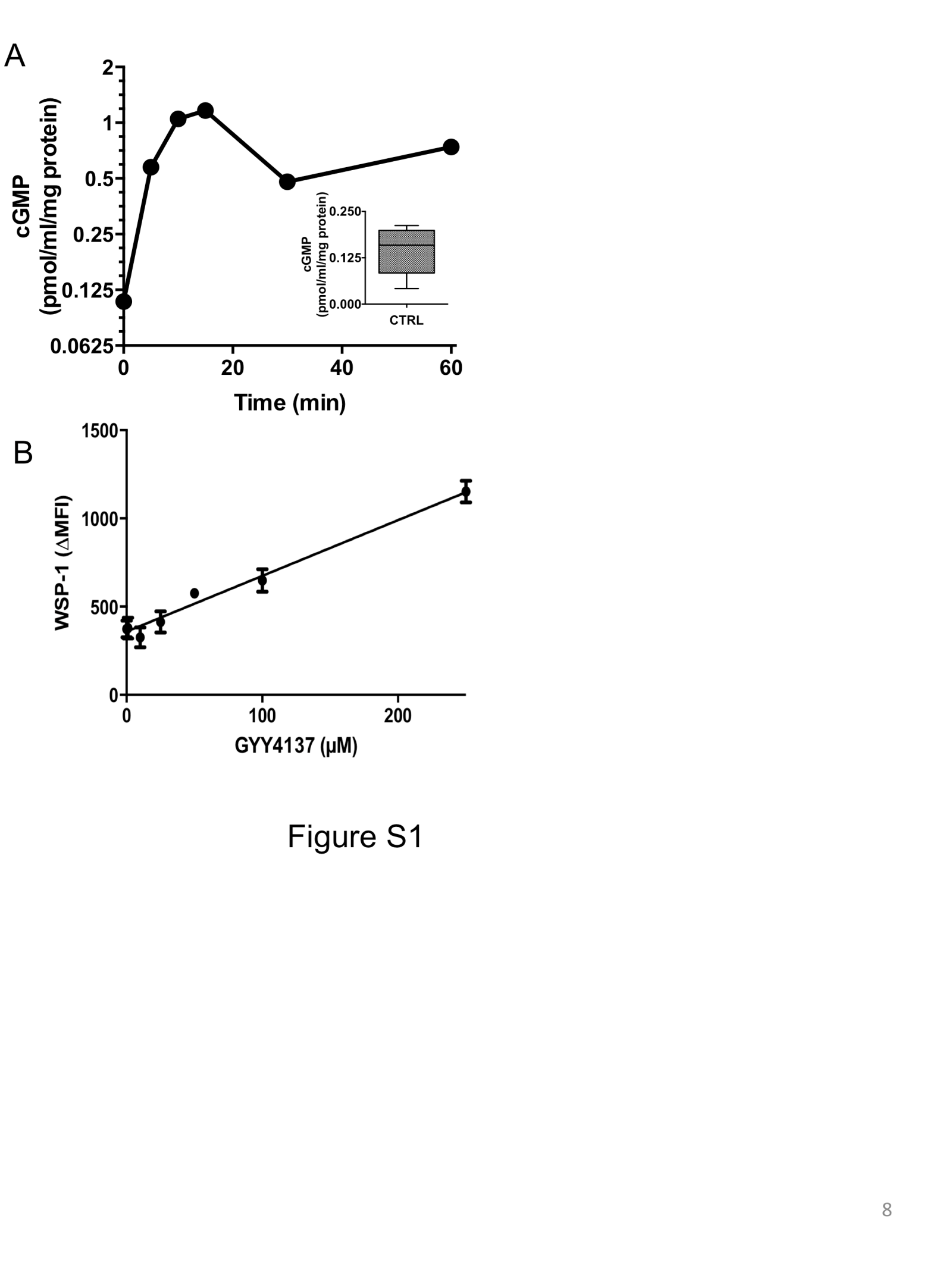
**

**Figure S1:** (**A**) Representative kinetics of cGMP accumulation in RFL-6 cells after stimulation with 100 µM SNAP. Inset: variability in intracellular cGMP concentration of cells in different passages. (**B**) Increase in intracellular WSP-1 fluorescence after 45 min of incubation with the slow sulfide donor GYY4137 as assessed by flow cytometry (n = 2).

**Figure S2:**

**
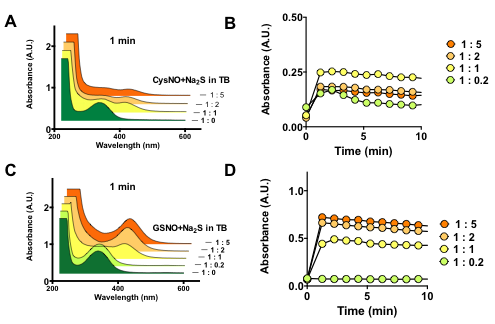
**

**Figure S2**: **Formation of SSNO^-^ from CysNO and GSNO in aqueous conditions.** Reaction of CysNO (1 mM) (**A,B**) or GSNO (**C,D**) with different concentrations of Na_2_S (0.2; 1; 2; or 5 mM) and in TRIS buffer 1M (TB) at pH 7.4. (**A,C**) UV-visible spectra of the different concentration ratios taken after 1 min of incubation. Green spectra, starting spectrum of CysNO (A) or GSNO (C). (**B,D**) Kinetics of formation of SSNO^-^ (λmax 412 nm ) from CysNO (B) and GSNO (D).

**Figure S3:**


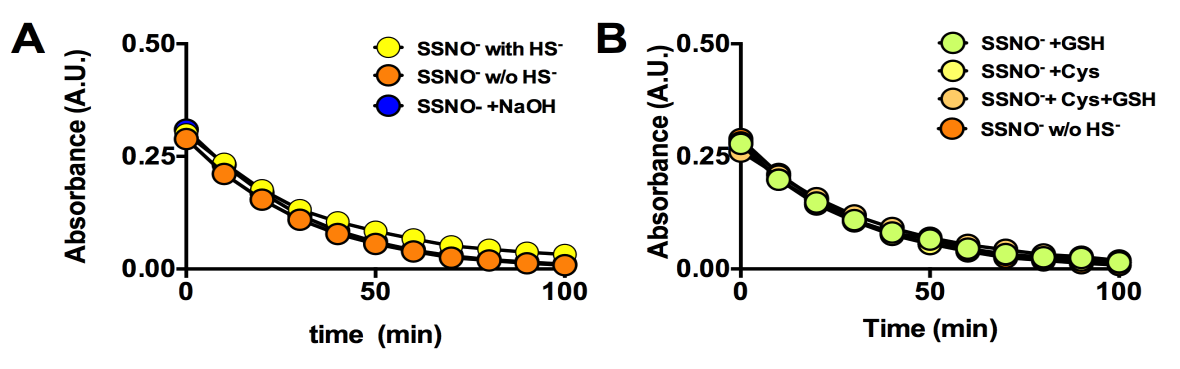


**Figure S3. Stability of SSNO^-^ in the presence of thiols and NaOH.** A SNAP/Na_2_S mixture (1:10) was incubated for 10 min in TRIS 1M at pH 7.4; an aliquot was bubbled for 5 min with nitrogen to remove excess sulfide (w/o HS^-^) and aliquot was kept at RT (with HS^-^). Removal of excess sulfide was checked by UV-visible spectroscopy (λ_max_ =250 nm). Thiols or NaOH were added at 1 mM final concentrations after sulfide removal. (**A,B**) Kinetics of decomposition of SSNO^-^ (λ_max_ 412 nm) in the presence or absence of excess sulfide (HS^-^), or after removal of sulfide by gassing the solution with N_2_ for 5 min.

**Figure S4:**


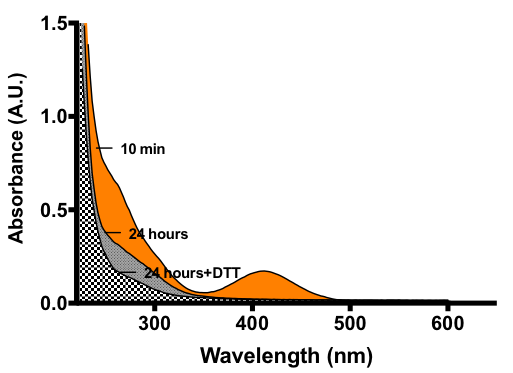


**Figure S4. SSNO^-^ forms polysulfides upon decomposition.** UV-visible spectra of a SNAP/Na_2_S mixtures (1:10) in TRIS 1M at pH 7.4 after 10 min (orange) or 24 h (grey) of incubation at RT in the dark. After addition of 1 mM dithiotheritol (DTT) the broad absorption band at 250-310 nm disappears, confirming the sulfane sufur (polysulfide) nature of this spectral feature.
